# Intact Acquisition and Short-Term Retention of Non-Motor Procedural Learning in Parkinson’s Disease

**DOI:** 10.1371/journal.pone.0149224

**Published:** 2016-02-23

**Authors:** Muriel T. N. Panouillères, George K. Tofaris, Peter Brown, Ned Jenkinson

**Affiliations:** 1 Nuffield Department of Clinical Neurosciences, University of Oxford, John Radcliffe Hospital, Oxford, United Kingdom; 2 School of Sport, Exercise and Rehabilitation Sciences, University of Birmingham, Birmingham, United Kingdom; Cardiff University, UNITED KINGDOM

## Abstract

Procedural learning is a form of memory where people implicitly acquire a skill through repeated practice. People with Parkinson’s disease (PD) have been found to acquire motor adaptation, a form of motor procedural learning, similarly to healthy older adults but they have deficits in long-term retention. A similar pattern of normal learning on initial exposure with a deficit in retention seen on subsequent days has also been seen in mirror-reading, a form of non-motor procedural learning. It is a well-studied fact that disrupting sleep will impair the consolidation of procedural memories. Given the prevalence of sleep disturbances in PD, the lack of retention on following days seen in these studies could simply be a side effect of this well-known symptom of PD. Because of this, we wondered whether people with PD would present with deficits in the short-term retention of a non-motor procedural learning task, when the test of retention was done the same day as the initial exposure. The aim of the present study was then to investigate acquisition and retention in the immediate short term of cognitive procedural learning using the mirror-reading task in people with PD. This task involved two conditions: one where triads of mirror-inverted words were always new that allowed assessing the learning of mirror-reading skill and another one where some of the triads were presented repeatedly during the experiment that allowed assessing the word-specific learning. People with PD both ON and OFF their normal medication were compared to healthy older adults and young adults. Participants were re-tested 50 minutes break after initial exposure to probe for short-term retention. The results of this study show that all groups of participants acquired and retained the two skills (mirror-reading and word-specific) similarly. These results suggest that neither healthy ageing nor the degeneration within the basal ganglia that occurs in PD does affect the mechanisms that underpin the acquisition of these new non-motor procedural learning skills and their short-term memories.

## Introduction

Procedural learning is a form of implicit memory, where people are exposed to information and unconsciously acquire motor or cognitive skills simply through practice [1]. Acquisition of such skills is visible by an increased accuracy and/or speed due to the repeated exposure of a specific procedure. This implicit form of memory is often contrasted with the declarative memory that corresponds to the conscious acquisition of events, facts or skills. As people with Huntington’s disease or Parkinson’s disease can be impaired on various implicit skill acquisition tasks, this suggests a critical involvement of basal ganglia in procedural learning [2,3].

Procedural learning and memory can be decomposed into the acquisition phase during which the skill is gained and the retention/consolidation phase, where the skill is partially, completely maintained or even enhanced over a period of time before retesting. Different types of task have been used to assess this procedural learning (including its acquisition and retention) in people with Parkinson’s disease. The most common form of motor procedural learning is the Serial Reaction Time Task (SRTT) [4], where participants are required to respond as quickly as possible to visual stimuli that are presented in a repeating sequence (however, if the sequence is long enough participants are not explicitly aware of the sequence). Despite some controversies, it is well accepted that people at a moderately severe stage of PD suffers from deficits in acquisition of the SRTT, as their improvement in response time is less than the one of aged-matched controls [5–10]. Another form of motor procedural learning that has been studied in people with PD is motor adaptation. To investigate and induce motor adaptation, participants are presented with a perturbed visual feedback and they progressively and unconsciously learn to correct for this visual perturbation when repeated [11]. It has recently been shown that although people with Parkinson’s disease acquire this motor skill similarly to control participants, they do not show a normal retention of the task in the long-term [12–14]. Indeed, people with PD improved their accuracy in the task as well as age-matched control participants when they were first exposed to this task. However, when people with PD were re-tested 24 to 48hrs later, they do not show any benefit from having performed a first learning session, contrary to control participants who re-learnt the task faster and/or started the follow-up sessions with higher accuracies than the first one. Thus, depending of the type of motor procedural learning, people with Parkinson’s disease can be impaired either in the acquisition phase (SRTT) or in the retention phase (motor adaptation). However, people with Parkinson’s disease are known to have disrupted sleep patterns [15,16]. Because sleep plays a crucial role in the memory retention process [17], the retention deficits highlighted in the motor adaptation task could be the results of sleep impairments rather than due to Parkinson’s disease itself. A recent study assessed short-term retention of motor adaptation by testing people with Parkinson’s disease twice with only a 50-minutes break between the two learning sessions [18]. The authors then found that people with Parkinson’s disease were impaired in this short-term retention test, and this then suggests that deficits in short-term memory (and long-term memory) could be a characteristic feature of Parkinson’s disease.

Parkinson’s disease is a neurodegenerative disease with primary symptoms in the motor domain. It is possible that the deficits in the acquisition and short-term retention of procedural learning in the motor domain described above are due to well-known impairment of the motor system. However, it might be that the deficit is due to a more general deficit in procedural learning beyond the motor system. Mirror-reading represents a more cognitive procedural learning task where participants are repeatedly exposed to words presented as if they were seen in a mirror. Thanks to the repeated exposure to mirror-inverted words, participants progressively learn to perceptively recognise the letters and consequently they are able to perform the mirror-reading faster with practice. This task usually contains words that are repeated over the course of the experiment that allows assessment of “word-specific” learning, where a benefit in reading time is induced by repeated priming with the same words. Analogous with motor adaptation, most studies investigating mirror-reading in people with PD found that they acquire the mirror-reading skill and the repeated-words similarly to control participants [19–23](but see [24] for opposite results). However, it was found that the retention of this cognitive learning (both mirror-reading and word-specific) in the long-term was impaired in people with Parkinson’s disease [19,22,24]. Indeed, when people with PD were retested on the mirror-reading task one day, a few days or 3 months following the first exposure to the task, their performance was worse compared to that of age-matched control participants. However, these deficits could again be caused by disrupted sleep. We speculated that because the pattern of results on the mirror-reading learning task is highly similar to the pattern of the motor adaptation task (no deficit in acquisition, deficit in long-term retention in people with PD), that people with PD will suffer from short-term retention deficits in this non-motor procedural learning task.

Therefore, the main aim of this study was to investigate the short-term retention of the mirror-reading task in people with Parkinson’s disease. We compared the performance of people with PD to age-matched controls two sessions of mirror-reading with a short break of 50 minutes in-between. Because of the role on dopamine in reinforcement learning and automatization processes [25], we also considered whether the dopamine medication would have an influence on the acquisition of the task or the short-term retention and thus tested two groups of participants either ON or OFF their normal medication. Finally, we also assessed the performance of a group of young participants in this study to evaluate the effect of healthy and pathological ageing on the non-motor procedural learning. In the mirror-reading task, some words were non-repeated across blocks and this allows evaluating the ability to acquire the rules or procedures necessary for mirror-reading, while some words were repeated across blocks and this allows testing the word-specific learning and thus evaluating the ability to benefit from frequent repetition of specific words.

## Materials and Methods

### Subjects

Twenty-six people with idiopathic Parkinson’s disease were recruited. The only two inclusion criteria for this study were that the people were diagnosed with idiopathic Parkinson’s disease and that they were able to perform the mirror-reading task. One person was excluded as they were diagnosed afterwards to have a mutation in the Parkin gene, another was excluded due to excessive dyskinetic symptoms on the day of testing and one was excluded as their performance of the reading task was an outlier in the group analysis; outside the range of the group mean ± 2 standard deviation. In total, we included 11 people with Parkinson’s disease on their normal medication (PD ON—mean age: 64.1 ±5 years old, 5 females, all right-handed, mean motor assessment UPDRS: 32.1 ±12.6, mean disease duration: 5.9 ±5 years) and 12 people with Parkinson’s disease tested at least 12 hours after last taking their medication (PD OFF—mean age: 64.3 ±6 years old, 5 females, all right-handed, mean motor assessment UPDRS: 35.7 ±7.3, mean disease duration: 4.6 ±2.7 years). People with PD were randomly assigned to one group or the other. People in the OFF group were asked to not take any Parkinson’s disease related medication in the 12 hours preceding the study.

Fourteen healthy older adults with no history of neurological disorder were recruited for this study. Data of one subject were excluded as performance for this subject in the reading task was an outlier in the group analysis (see above) of the older group. Thus, the data of 13 older subjects are presented in this study (mean age: 63.1 ±7.3 years old, range: [52–76], 6 females, all right-handed). Fourteen healthy young adults were recruited to the study, again data of one subject were removed as it was an outlier in the group. In summary, the data of only 13 young subjects were included (mean age: 33.1 ±8.7 years old, range: [22–49], 6 females, two left-handed participants). Young and older healthy participants had no history of neurological disorder.

All participants were native English speakers and had normal or corrected to normal vision. The reading abilities of the participants were assessed using the Schonell Reading Test [26]. This test evaluated the pronunciation of 100 words and it was used to avoid the confound that the participants may be slower in reading the mirror-inverted words because they did not have good reading abilities and so did not know the presented stimuli.

### Ethics statement

All experimental procedures conformed to the Code of Ethics of the World Medical Association (Declaration of Helsinki) and were approved by the National Research Ethics Service (NRES) Committee South Central—Oxford B and C. Written informed consents were obtained from all participants before inclusion in the present project.

### Materials and procedures

**Fig 1:**
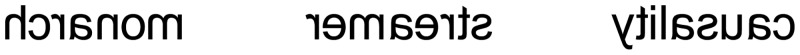
An example of a mirror-inverted triad of words.

PsychoPy presentation software [27], was use to display visual stimuli on a 40 × 30 cm computer monitor and recording participants’ reading times. Words were chosen from the Medical Research Council (MRC) psycholinguistic database, using the MRC Machine usable dictionary (Version 2.0). Inclusion criteria for the words were: nouns of 7 to 9 letters long, composed of 2 to 4 syllables with a printed familiarity rating going from 300 to 500. Word familiarity reflects how commonly a word is experienced. In the MRC psycholinguistic database, printed familiarity values lie in the range 100 to 700, with a mean of 488 and a standard-deviation of 99. A word with a low value of familiarity means that subjects will not have experienced this word very often. Words containing the letters b, d, p or q were excluded from this study to avoid confounding them with their non-mirrored version. Visual stimuli consisted of a triad of mirrored white words on a grey background, in Backwards font (equivalent of Arial font, but presented backward, in order to achieve a mirror presentation of the words). The word triads subtended a horizontal angle of 7–8° on either side of the fixation cross, with the subjects sitting 75 cm away from the screen (Fig 1).

Behavioural testing was divided in two phases separated by a 50-minute break. Each phase was identical and composed of 5 blocks of 10 trials each, with 60 seconds rest between blocks. Each block contained 5 non-repeated triads and 5 triads that were repeated in all the blocks. Repeated and non-repeated triads were randomised across blocks. Familiarity between the repeated words and the non-repeated ones in each of the 10 blocks did not differ (univariate ANOVA with mean familiarity of each triad with the factor “Repeated words” versus “non-repeated words”: F[10,154]<1, p = 1). Each trial started with a blank screen for 2-seconds followed by a fixation cross presented in the centre of the screen for 500 ms. The disappearance of the fixation cross was concomitant with the presentation of a triad of mirror-inverted words on the horizontal meridian of the screen. Subjects were required to read aloud the triads of mirror-inverted words as fast and accurately as possible, starting from the rightmost word. Reading time was the time taken from the appearance of the triad on screen until the last syllable of the third word was uttered. The time to read the triplet was recorded by the experimenter pressing a response key once the last word was correctly read by the participant. The experimenter rather than the participant pressed the spacebar for two reasons: first, in case participants made mistakes, they had an opportunity to correct before the experimenter recorded the time when all three words were read correctly; second, because Parkinson’s disease is characterised by a slowing in initiating movement, slowness in pressing the response key could have been a potential confound with our measure of the reading time.

### Analysis

For all subjects, the reading time of each block in the first and second phase was averaged, separately for the repeated and non-repeated triads of words. Statistical analyses were performed with the SPSS Statistics software package (IBM, Armonk NY, USA) on the log-transformed reading times in order to perform parametric analysis [19]. The performance of all four groups was compared using separate repeated-measures ANOVAs for the non-repeated and repeated triads with phase and blocks as within-subject factors and groups as between-subject factor. The amount of learning that occurred during the first phase was calculated as the difference of the log-transformed reading time between the 5^th^ block of the first phase and the 1^st^ block of the first phase. Similarly, the amount of learning that occurred during the experiment was computed as the difference of the log-transformed reading time between the last block of the experiment and the 1^st^ block of the first phase. This amount of learning was compared across groups using separate ANOVAs for repeated and non-repeated triads with phase as within-subject factor and group as between-subject factor. The forgetting during the 50-minutes break was calculated separately for the non-repeated and repeated triads of words as the difference between the log-transformed reading time in block 6 and the log-transformed reading time in block 5. This forgetting was compared across groups using separate ANOVAs for repeated and non-repeated triads using group as between-subject factor. Significant ANOVAs were followed by Bonferroni post-hoc pairwise comparisons. In all cases significance was set at p<0.05.

## Results

### Participants’ characteristics

The demographics of the people with Parkinson’s disease are presented Table 1. The two groups of people with Parkinson’s disease, ON and OFF medication, did not differ in respect to their symptoms (UPDRS motor assessment: t[16] = -0.82, p = 0.42) and disease duration (t[21] = 0.82, p = 0.42). The reading abilities of the participants were assessed using the Schonell reading test and they did not differ between the 4 groups of participants (F[3,45]<1, p = 0.51). Thus, our 4 groups of participants possessed the same reading capabilities suggesting that any difference in the mirror-reading task among the groups will not be accounted for by pre-existing differences in reading ability.

**Table 1:** Characteristics of the people with Parkinson’s disease.

Patient	Group	Age (years)	Sex	Handedness	UPDRS Part III	Disease Duration (years)	Schonell (year)
P1	PD-ON	58	M	Right	49	4	15
P2	PD-ON	54	M	Right	46	1	15
P3	PD-ON	65	M	Right	44	12	14.9
P4	PD-ON	69	M	Right	31	10	14.8
P5	PD-ON	66	M	Right	28	2	14.8
P6	PD-ON	69	F	Right	12	3	14.8
P7	PD-ON	61	F	Right	40	17	14.7
P8	PD-ON	71	M	Right	40	3	14.7
P9	PD-ON	66	F	Right	24	5	14.7
P10	PD-ON	63	F	Right	15	3	14.8
P11	PD-ON	63	F	Right	24	5	14.8
P12	PD-OFF	53	F	Right	40	9	14.9
P13	PD-OFF	64	F	Right	31	5	14.8
P14	PD-OFF	66	M	Right	51	5	14.7
P15	PD-OFF	65	M	Right	32	6	14.8
P16	PD-OFF	75	F	Right	31	3	14.8
P17	PD-OFF	55	M	Right	30	0.75	15
P18	PD-OFF	61	M	Right	48	3	15
P19	PD-OFF	74	M	Right	30	1	14.8
P20	PD-OFF	66	F	Right	39	3	14.8
P21	PD-OFF	65	F	Right	32	6	14.9
P22	PD-OFF	67	M	Right	32	4	15
P23	PD-OFF	61	M	Right	32	9	14.8

UPDRS: Unified Parkinson’s Disease Rating Scale—Part III corresponds to the motor part of the scale.

### Performance for the non-repeated triads

The reading times for the non-repeated triads for the people with Parkinson’s disease ON their medication (PD ON), the people with Parkinson’s disease OFF their medication (PD OFF), the healthy older adults and young adults are presented in Table 2. The reading times were averaged across the different blocks before (Phase 1, blocks 1 to 5) and after (Phase 2, blocks 6 to 10) the 50-minutes break. The reduction across blocks in the mean reading time for the non-repeated triads is considered as reflecting the magnitude of acquisition of the mirror-reading skill.

**Table 2:** Mean reading time in seconds for the non-repeated triads of all 10 blocks.

	Phase 1					Phase 2				
Blocks	1	2	3	4	5	6	7	8	9	10
Young	11.40	8.56	8.19	7.72	9.32	7.79	7.62	8.17	8.81	8.37
Older	22.89	16.79	14.94	13.96	17.83	16.23	14.89	14.29	15.34	13.26
PD ON	21.98	14.51	13.93	14.76	17.15	14.31	15.84	15.07	14.02	12.78
PD OFF	19.00	13.81	13.48	13.41	14.67	16.12	14.73	16.13	16.073	14.14

**Fig 2:**
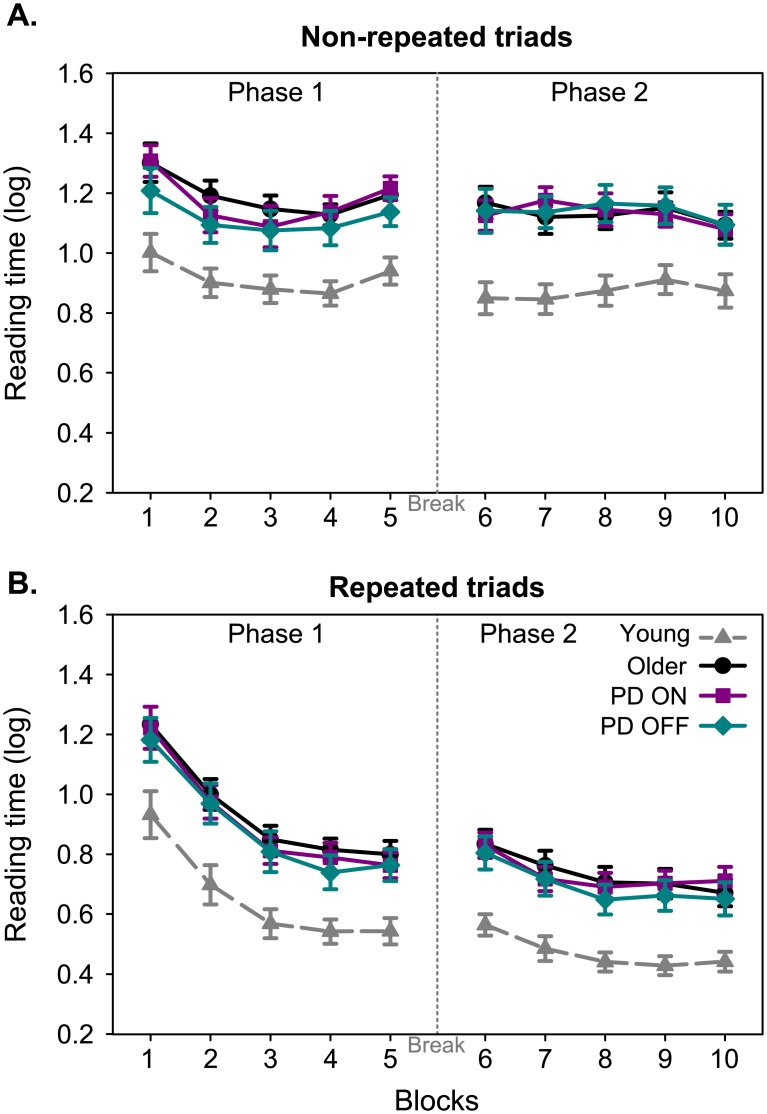
Mean reading times necessary for the participants to read the non-repeated triads (A) and the repeated triads (B) of mirrored-inverted words. The mean reading time (in seconds, log-transformed) was plotted as a function of the experimental blocks (1 to 10) for the young adults (grey), the older adults (black), the PD ON their medication (purple) and the PD OFF their medication (cyan). The blocks 6 to 10 were performed following a 50-minutes break represented by the dashed vertical line. Error bars are standard-error of the mean.

An improvement in reading time occurred for all subjects (Fig 2A) as they practiced the mirror reading task, visible by the progressive decrease in reading time across the blocks (Block effect: F[4,158] = 13.56, p<0.001). All groups improved their reading speed more during the first exposure to the mirror-reading task than during the second one that followed the 50-minutes break (Phase effect: F[1,158] = 21.48, p<0.001; Phase and Block interaction: F[4,158] = 26.67, p<0.001). Throughout the entire experiment, the only group difference was due to the young adults reading faster than older adults (Group effect: F[3,45] = 6.75, p<0.001; Bonferroni post-hoc comparisons: young vs PD OFF p<0.05; young vs older adults and young vs PD ON: p<0.01). The reading time of people with PD both ON and OFF their medication was matched to that one of older adults (Bonferroni post-hoc comparisons: p = 1).

For each participant, we calculated the amount of learning during the first phase as the reduction in reading time between the 5^th^ (last) block and the 1^st^ block in phase one. Similarly, we calculated the amount of learning across the entire experiment as the reduction in reading time between the 10^th^ block (last block in phase 2) and the 1^st^ block in phase 1. The amount of learning in the first phase and throughout the entire experiment was similar across all 4 groups (Group effect: F[3,45] = 1.07, p = 0.3.7; Group × Phase interaction: F[3,45] = 2.18, p = 0.10). Moreover, the learning achieved at the end of the experiment was stronger than at the end of the first phase (Phase effect: F[1,45] = 40.97, p<0.001). This then suggests that all groups of participants acquired the mirror-reading skill to a similar extent. Thus, people with PD ON and OFF their medication acquired it similarly than young and older adults.

Finally, we assessed how much of the mirror-reading skill was retained across the 50-minutes break by calculating the difference in reading time between the last block before the break and the first one following the break. This difference was compared across the four groups using a univariate ANOVA with the factor Group. No main effect of Group was detected (F[3,45] = 1.81, p = 0.16). A summary of the results for the non-repeated words is presented in S1 Table.

Thus, we found that the young adults performed the mirror-reading task much faster than all the three groups of older participants. However, it is important to note that the amount of learning of the mirror-reading skill was similar for all 4 groups of participants and that the people with PD acquired and retained the mirror-reading task similarly as healthy older adults.

### Performance for the repeated triads

The reading times for the repeated triads of mirror-inverted words for the people with Parkinson’s disease ON their medication (PD ON), the people with Parkinson’s disease OFF their medication (PD OFF), the healthy older adults and young adults are presented in Table 3. Repeating triads of words allows evaluating the ability to benefit from frequent repetition of specific words.

**Table 3:** Mean reading time in seconds for the repeated triads of all 10 blocks.

	Phase 1					Phase 2				
Blocks	1	2	3	4	5	6	7	8	9	10
Young	10.55	5.81	4.01	3.69	3.76	3.82	3.24	2.86	2.77	2.87
Older	19.01	10.90	7.58	6.84	6.73	7.38	6.34	5.68	5.52	5.02
PD ON	19.11	10.27	6.88	6.63	6.10	7.15	5.45	5.24	5.33	5.48
PD OFF	17.58	10.62	7.37	6.04	6.27	6.92	5.71	4.78	4.95	4.94

We expected that due to the repeated exposure to the repeated triads, participants become increasingly faster during learning. This should be visible as a larger decrease in reading time for repeated triads compared to novel triads due to the memory of the previous exposure to the repeated triplet (Fig 2B). All groups of participants progressively learnt the repeated triads as is visible by a progressive decrease in the reading time across blocks (Block effect: F[4,124] = 339.52, p<0.001). Performance improved across both phases of the task, but was more pronounced in the first phase, when subjects were first exposed to the repeated triads (Phase effect: F[1,124] = 272.99, p<0.001; Block × Phase interaction: F[4,124] = 127.14, p<0.001). Note that in this repeated triad condition, the improvement in reading time was larger (Fig 2B) than that for the non-repeated triads (Fig 2A), clearly showing the benefit for all participants of repeated exposure to the word-triplets. The only difference between groups for the repeated triads came from the young adults being quicker in reading the repeated words than the three groups of older participants (Group effect: F[3,45] = 7.63, p<0.001; Bonferroni post-hoc comparisons young vs PD ON and young vs PD OFF: p<0.01; young vs older adults: p<0.001). The performance of PD ON and OFF their normal medication matched that of the healthy older adults (Bonferroni post-hoc comparisons: p = 1).

For each participant, we then calculated the amount of learning during the first phase of the experiment as the reduction in reading time between the 5^th^ (last) block and the 1^st^ block of phase one. Similarly, we also calculated the amount of learning across the entire experiment as the reduction in reading time between the 10^th^ block and the 1^st^ block. The reduction in reading time for the repeated triads in the first phase and throughout the entire experiment was similar across all 4 groups (Group effect: F[3,45]<1, p = 0.79; Group × Phase interaction: F[3,45] = 1.87, p = 0.15). All groups were again quicker in the last block of the experiment than they were in the last block of the first phase (Phase effect: F[1,45] = 69.02, p<0.001). Thus, repeating triads of words over the course of the experiment benefitted all participants in the same way, as they all decreased their reading times by a similar amount.

Finally, we assessed how the repeated triads of words were retained during the 50-minutes by calculating the difference in reading time between the last block before the break and the first block following the break. Retention did not significantly differ across group (Group effect: F[3,45]<1, p = 0.53). A summary of the results for the repeated words is presented in S1 Table.

Thus, despite the young adults again reading faster than the older adults, all participants were able to acquire the repeated triads of words similarly. Moreover, all groups of participants retained the repeated triads of words similarly.

## Discussion

The present study was aimed at investigating the acquisition and short-term retention of a non-motor procedural learning in people with Parkinson’s disease. This was tested in two different conditions where words were either always new words (learning of the mirror-reading skill) or repeated (“word-specific” learning). We found that people with PD ON and OFF their medication, older adults and young adults all acquired the mirror-reading skill for non-repeated and repeated triads and this acquisition for the two conditions was similar across the four groups. This was despite the fact that the young adults have faster initial reading speed. We also found that the retention of both types of learning was similar across the four groups.

Young adults were faster in reading the mirror-inverted words than any of the older adults groups. This cannot be accounted by differences in reading capabilities, as all four groups of participants possessed the same reading abilities, but this is in agreement with studies showing that ageing is associated with slower reaction time and movement time [28]. Because ageing is associated with changes in both the cognitive and motor domains, the fact that older adults were slower than young adults in the mirror-reading task could be due to changes in any of these two domains. Despite this difference in initial performance, the amount of learning of both the mirror-reading skill and of the repeated triads was similar in all groups. The similar amount of learning suggests that healthy ageing and Parkinson’s disease retain the mechanisms that underpin the acquisition of these new skill and memories to the same extent. For the motor procedural learning, different results have been found depending of the task. In the case of the serial reaction time task (SRTT), healthy adults have been found to perform similarly than young adults (despite differences in initial performance) [29–32]. However, in the case of motor adaptation, it has been shown that, although initial performance is similar between young and older adults, the amount of learning in older adults is impaired relative to the one of young participants [33–41]. Because our reading task and the SRTT improvements rely on response time, while the motor adaptation task relies on the accuracy of the movement, it could be suggested that older adults can improve their response time to the same extent as young adults, but not their accuracy.

In agreement with most studies investigating mirror-reading of non-repeated and repeated words in people with Parkinson’s disease, we found that the acquisition of this new procedural skill was similar to healthy older adults [19–23] (but see [24] for contradictory results). These results are similar to those seen in motor adaptation, a form of motor procedural learning, where people with PD perform similarly as healthy older adults. Moreover, another similar feature between motor adaptation and mirror-reading is that long-term retention of both skills tested after a period of one day and up to 3 months after the first exposure is impaired in people with Parkinson’s disease [19,22,24].

However, the patterns of deficits in motor and non-motor procedural learning are not identical. Indeed, in the case of motor adaptation, it has been shown that patients with PD are impaired in their short-term retention [18]. Contrarily, in the case of mirror-reading, we found that the performance of the people affected by Parkinson’s disease after a short break (50 minutes) was identical to that of older control participants. The present study therefore suggests that Parkinson’s disease does not have a negative impact on the short-term (i.e. less than one day) retention of new non-motor procedural skills, though further investigations should establish if the deficits in retention after 1 day are directly due to the pathology of Parkinson’s disease itself or secondary to disrupted sleep. Moreover, it appears that basal ganglia function may be more essential for the retention of procedural learning in the motor domain than in the non-motor domain.

In the present study, we report that people with Parkinson’s disease do not differ from older adults in their learning and retention of the mirror-reading task. Previous studies have also shown that people with PD have a similar capacity for many cognitive skills as healthy older adults such as the verbal serial reaction time task, artificial grammar [42], the semantic categorization [43] and the Tower of London task [44]. All these results suggest that most learning processes are preserved in Parkinson’s disease. In the present study, it is possible that the people with PD did not show deficits in the acquisition and retention in mirror-reading because they had relatively mild PD (see [10] for similar conclusion on the SRTT). However, another explanation could be that some compensatory mechanisms would develop in people with PD to counter-act the effect of the disease. For example, some procedural learning tasks have been shown to activate in people with PD the neural substrates of declarative memory [44,45], that could allow to compensate for deficits. Further studies will then be needed in order to disentangle between these two possibilities.

## Conclusions

People with Parkinson’s disease had no deficit compared to healthy older adults in their ability to acquire and retain—in the short-term—the new skill necessary to be able to read mirror-inverted words. They also demonstrated a similar benefit of repeated exposure to some of the triads of words across the experiment. Reading performance of the older adults (PD ON, PD OFF and healthy older adults) was slower relative to that the of the young adults. Our results suggest that unlike some forms of motor procedural learning, a mild form of Parkinson’s disease does not have an impact on the acquisition and short-term retention of non-motor procedural learning.

## Supporting Information

S1 Dataset Individual Reading time for each block and subjects details. (XLSX)

S1 Table summary of statistical results for the non-repeated and repeated words. (DOCX)
