# Supplementary material for: Intact Acquisition and Short-Term Retention of Non-Motor Procedural Learning in Parkinson’s Disease
Source: PLoS One. 2016 Feb 23;11(2):e0149224. doi: 10.1371/journal.pone.0149224 (PMC4764369; doi:10.1371/journal.pone.0149224)
Supplement: S1 Table — (DOCX) [file pone.0149224.s002.docx]

**Supporting Table**

|  | Reading speed | Learning | Retention |
| --- | --- | --- | --- |
| Non-repeated words | Young adults faster than all 3 older adults group | Amount of learning similar across all 4 groups | Retention during 50-min break similar across all 4 groups |
| Repeated words | Young adults faster than all 3 older adults group | Amount of learning similar across all 4 groups | Retention during 50-min break similar across all 4 groups |
